# Supplementary material for: Content-rich biological network constructed by mining PubMed abstracts
Source: BMC Bioinformatics. 2004 Oct 8;5:147. doi: 10.1186/1471-2105-5-147 (PMC528731; doi:10.1186/1471-2105-5-147)
Supplement: Additional File 2 — The original results of the above study (non-essential files are deleted to keep the file size under the limit set by BMC bioinformatics). [file 1471-2105-5-147-S2.bz2 › chilibotAdditionalFile2/dip40/622ID10417423E11008/html/CAPN2_CAPNS1.html]

 


 **CAPN2** and **CAPNS1** 
  
Found 230 abstracts in PubMed, retrieved 40.  
 

 What does Google say? 
 PDF only 
| .edu only 

---

**Interactive relationship** (e.g. stimulation, inhibition, etc)

**Neutral relationship**- 7 In order to determine which domain of the calpain I large subunit was involved in the interaction with these real clones, the 37 clones were transformed into AH109 pGBKT7 CANP  [ **CAPNS1** ]   [ **CAPN2** ]  II, AH109 pGBKT7 CANP  [ **CAPNS1** ]   [ **CAPN2** ]  III or AH109 pGBKT7 CANP  [ **CAPNS1** ]   [ **CAPN2** ]  IV.  Ref: 12358155 Hypertens Res, 2002
- m Calpain  [ **CAPN2** ]  calcium activated neutral proteinase  [ **CAPNS1** ]   [ **CAPN2** ]  in Alzheimer s disease brains.  Ref: 9654354 Neurosci Lett, 1998
- Thus, we successfully constructed 4 bait expression vectors, pGBKT7 CANP  [ **CAPNS1** ]   [ **CAPN2** ] , pGBKT7 CANP  [ **CAPNS1** ]   [ **CAPN2** ]  II, pGBKT7 CANP  [ **CAPNS1** ]   [ **CAPN2** ]  III and pGBKT7 CANP  [ **CAPNS1** ]   [ **CAPN2** ]  IV, and obtained 37 real positive clones that interacted with the calpain I large subunit by screening a human heart cDNA library using pGBKT7 CANP  [ **CAPNS1** ]   [ **CAPN2** ]  as bait.  Ref: 12358155 Hypertens Res, 2002

**Non-interactive relationship** (e.g. studied together, co-existance, homology, etc.)

- calcium chelator, A23187 calcium ionophore, EGTA plus A23187 extra intra cellular calcium chelation, trifluoperazine and CGS9343B calmodulin inhibitors, and E 64 CANP  [ **CAPNS1** ]   [ **CAPN2** ]  inhibitor at the time of the natural peak of melatonin release.  Ref: 9605476 Neurosci Lett, 1998
- An expedient method for the purification of a calcium activated neutral protease CANP  [ **CAPNS1** ]   [ **CAPN2** ]  of the goat uterus has been designed.  Ref: 10432223 Mol Cell Endocrinol, 1999
- Proteases such as calcium activated neutral proteinase  [ **CAPNS1** ]   [ **CAPN2** ]  calpain have been implicated in axon and myelin destruction following injury since they degrade structural proteins in the axon myelin unit.  Ref: 10878587 J Neurosci Res, 2000
- By employing this method, CANP  [ **CAPNS1** ]   [ **CAPN2** ]  activity in different regions of rat brain was determined.  Ref: 12385765 J Neurosci Methods, 2002
- In order to investigate the role of calcium activated neutral proteinase  [ **CAPNS1** ]   [ **CAPN2** ]  calpain, which degrades myelin proteins, the activity and expression translational and transcriptional of this enzyme were examined in spinal cords of Lewis rats with experimental allergic encephalomyelitis EAE, an animal model of MS.  Ref: 9630523 Brain Res, 1998
- Proteolytic enzyme expression was studied by matrix metalloproteinases MMP immunoreactivity IR in muscle biopsies from patients with amyotrophic lateral sclerosis ALS, spinal muscle atrophy SMA and chronic axonal neuropathies CANP  [ **CAPNS1** ]   [ **CAPN2** ] .  Ref: 10511442 Neuroreport, 1999
- Both mu and m calpains are heterodimers, consisting of a distinct large 80 kDa catalytic subunit, encoded by the genes Capn1 and  **Capn2** , and a common small 28 kDa regulatory subunit Capn4  [ **CAPNS1** ] .  Ref: 10825211 Mol Cell Biol, 2000
- Thus, proteases have been implicated in myelin protein degradation, and recent studies have demonstrated increased expression and activity of a calcium activated neutral proteinase  [ **CAPNS1** ]   [ **CAPN2** ]  calpain in experimental allergic encephalomyelitis, the corresponding animal model of MS.  Ref: 10500203 Proc Natl Acad Sci U S A, 1999
- C type natriuretic peptide and cANP  [ **CAPNS1** ]   [ **CAPN2** ]  4 23 revealed limited competition with radiolabeled ANP only during early lactation, indicating a more heterogeneous receptor population at that time.  Ref: 9564878 Endocrinology, 1998
- Effects of ethylene glycol tetraacetic acid, A23187 and calmodulin, calcium activated neutral proteinase  [ **CAPNS1** ]   [ **CAPN2** ]  antagonists on melatonin secretion in perifused chick pineal gland.  Ref: 9605476 Neurosci Lett, 1998
- The calcium activated neutral proteases CANP  [ **CAPNS1** ]   [ **CAPN2** ] , calpains have been implicated in both acute and chronic neurodegenerative processes.  Ref: 9473662 Brain Res Mol Brain Res, 1998
- In none of the 19 patients with LGMD, were extended deletions in the CANP  [ **CAPNS1** ]   [ **CAPN2** ]  3 gene found.  Ref: 10687099 Genetika, 1999
- Overall cytokine immunoreactivity was highest in VANP, less strong in CIDP and lowest in CANP  [ **CAPNS1** ]   [ **CAPN2** ] .  Ref: 12734666 Acta Neuropathol Berl , 2003
- 3 After 4 bait vectors were transformed into AH109 by the lithium acetate mediated method, AH109 pGBKT7 CANP  [ **CAPNS1** ]   [ **CAPN2** ] , AH109 pGBKT7 CANP  [ **CAPNS1** ]   [ **CAPN2** ]  II, AH109 pGBKT7 CANP  [ **CAPNS1** ]   [ **CAPN2** ]  III, and AH109 pGBKT7 CANP  [ **CAPNS1** ]   [ **CAPN2** ]  IV were obtained, respectively.  Ref: 12358155 Hypertens Res, 2002
- ANP, cANP  [ **CAPNS1** ]   [ **CAPN2** ]  4 23, and VIP initiated identical signaling cascades consisting of Ca2 influx, activation of eNOS via Gi1 and Gi2, stimulation of cGMP formation, and muscle relaxation.  Ref: 9843699 Am J Physiol, 1998
- ANP and cANP  [ **CAPNS1** ]   [ **CAPN2** ]  4 23 inhibited forskolin stimulated cAMP in a PTx sensitive fashion.  Ref: 10859228 Am J Physiol Gastrointest Liver Physiol, 2000
- it is also the major factor in the activation of the calcium activated neutral proteinase  [ **CAPNS1** ]   [ **CAPN2** ] , calpain.  Ref: 11585637 J Neuroimmunol, 2001
- This structure of exon 4 of the CANP  [ **CAPNS1** ]   [ **CAPN2** ]  3 gene was found neither in 16 patients with DBM who had deletions in the DMD gene nor in 16 patients with DBM who had no deletions in the DMD gene.  Ref: 10687099 Genetika, 1999
- Calpain, a calcium activated neutral proteinase  [ **CAPNS1** ]   [ **CAPN2** ] , has been implicated in myelin and cytoskeletal protein degradation following spinal cord injury.  Ref: 9878837 Brain Res, 1999
- Calcium activated neutral proteinase  [ **CAPNS1** ]   [ **CAPN2** ]  calpain is a ubiquitous, cytosolic endopeptidase which is believed to play a role in many neural functions.  Ref: 9710268 J Neurosci Res, 1998
- Calpain, also named CANP  [ **CAPNS1** ]   [ **CAPN2** ]  for calcium activated neutral protease, is an intracellular cytoplasmatic non lysosomal cysteine endopeptidase that requires calcium ions for activity.  Ref: 9988224 Int J Cancer, 1999
- SNP, but not PANP or CANP  [ **CAPNS1** ]   [ **CAPN2** ] , was associated with the intake of the neuropathogenic drugs dideoxycytidine, dideoxyinosine, and didehydro dideoxythymidine p < ; 0.05.  Ref: 10750639 Clin Auton Res, 2000
- A large subunit of calpain, a calcium activated neutral proteinase  [ **CAPNS1** ]   [ **CAPN2** ] , from Schistosoma japonicum was cloned and expressed in Escherichia coli.  Ref: 11119528 Infect Immun, 2001
- The mu and m calpains consist of a mu or m 80 kDa large subunit genes Capn1 and  **Capn2** , and a common 28 kDa small subunit Capn4  [ **CAPNS1** ] .  Ref: 11602605 J Biol Chem, 2001
- The purpose of this article is to examine the prevalence, degree, and natural course of pupillary neuropathy PANP, cardiovascular autonomic neuropathy CANP  [ **CAPNS1** ]   [ **CAPN2** ] , and sensorimotor neuropathy SNP and to study the impact of disease stage and medication on neuropathy in 61 consecutive patients with HIV.  Ref: 10750639 Clin Auton Res, 2000
- Calcium activated neutral proteinase  [ **CAPNS1** ]   [ **CAPN2** ]  calpain has been extensively studied over the past three decades such that many enzymatic and structural properties of this enzyme are well understood.  Ref: 10082076 J Neurosci Res, 1999
- To investigate potential therapeutic effects of atrial natriuretic peptide ANP gene delivery on renal and cardiac disorders, adenovirus harboring the human ANP gene Ad.RSV cANP  [ **CAPNS1** ]   [ **CAPN2** ]  was delivered into Dahl salt sensitive DSS rats on a high salt diet.  Ref: 9681414 Hum Gene Ther, 1998
- We used immunohistochemistry including double labeling and morphometry to localize and quantify the expression of interleukin 1 beta IL 1beta, IL 6, and tumor necrosis factor alpha TNF in sural nerve biopsy samples of 41 patients with vasculitic neuropathy VANP, chronic inflammatory demyelinating neuropathy CIDP, non inflammatory chronic axonal neuropathy CANP  [ **CAPNS1** ]   [ **CAPN2** ] , and 3 controls.  Ref: 12734666 Acta Neuropathol Berl , 2003
- In VANP and CANP  [ **CAPNS1** ]   [ **CAPN2** ] , a higher cytokine content was associated with neuropathic pain.  Ref: 12734666 Acta Neuropathol Berl , 2003
- Calcium activated neutral proteinase  [ **CAPNS1** ]   [ **CAPN2** ]  calpain is an endopeptidase present in the central nervous system which degrades myelin proteins.  Ref: 10212825 Histol Histopathol, 1999
- In most patients with LGMD, the amplification of the promoter region and exons,, 5, and 6 of the CANP  [ **CAPNS1** ]   [ **CAPN2** ]  3 gene yielded a single product of corresponding length, but in six patients, amplification of exon 4 of the CANP  [ **CAPNS1** ]   [ **CAPN2** ]  3 gene yielded two products of different size.  Ref: 10687099 Genetika, 1999
- The rate of gastric emptying was related to CANP  [ **CAPNS1** ]   [ **CAPN2** ]  but not gastric symptoms or actual glycemic control.  Ref: 12600185 Pol Arch Med Wewn, 2002
- Cloning and molecular characterization of calpain, a calcium activated neutral proteinase  [ **CAPNS1** ]   [ **CAPN2** ] , from different strains of Schistosoma japonicum.  Ref: 11227763 Parasitol Int, 2000
- 2 the descending axons are mainly peptidergic CRF, vasopressin, oxytocin, somatostatin, enkephalin, POMC, and cANP  [ **CAPNS1** ]   [ **CAPN2** ] , while the ascending fibers are both peptidergic enkephalin, NPY, neurotensin, dynorphins and catecholaminergic.  Ref: 10569279 Front Neuroendocrinol, 1999
- Recent molecular biological approaches indicate that calpain, also named CANP  [ **CAPNS1** ]   [ **CAPN2** ]  for calcium activated neutral protease and originally characterized as an intracellular cytoplasmatic nonlysosomal cysteine protease that requires calcium ions for activity, constitutes a large superfamily consisting of ubiquitous and tissue specific homologues, which are widely distributed in cells of various organisms from human to fungus.  Ref: 10403824 Biochem Biophys Res Commun, 1999
- Pretreatment of muscle cells with cANP  [ **CAPNS1** ]   [ **CAPN2** ]  4 23, a selective agonist of the natriuretic peptide clearance receptor, NPR C, which activates phospholipase C PLC beta3 via the betagamma subunits of G i 1 and G i 2, inhibited the PLC beta response to somatostatin and D Pen enkephalin.  Ref: 11003572 Am J Physiol Cell Physiol, 2000
- The enzyme is a heterodimer, encoded by the genes  **capn2** , for the large subunit, and capn4  [ **CAPNS1** ] , for the small subunit.  Ref: 12559913 J Mol Biol, 2003
- In binding studies using 125I labeled atrial natriuretic peptide 125I ANP and 125I VIP as radioligands, VIP, ANP, and the selective NPR C ligand cANP  [ **CAPNS1** ]   [ **CAPN2** ]  4 23 bound with high affinity to NPR C.  Ref: 9843699 Am J Physiol, 1998
- The utility of the method was evaluated by assaying the proteolytic activities of trypsin and calcium activated neutral protease CANP  [ **CAPNS1** ]   [ **CAPN2** ] .  Ref: 12385765 J Neurosci Methods, 2002
- Atrial natriuretic peptide ANP, the selective NPR C ligand cANP  [ **CAPNS1** ]   [ **CAPN2** ]  4 23, and vasoactive intestinal peptide VIP inhibited 125 I ANP and 125 I VIP binding to muscle membranes in a pattern indicating high affinity binding to NPR C.  Ref: 10859228 Am J Physiol Gastrointest Liver Physiol, 2000
- Autonomic and sensorimotor neuropathy are frequent in patients with HIV, and progression of CANP  [ **CAPNS1** ]   [ **CAPN2** ]  may put patients at risk for unexpected cardiorespiratory arrest.  Ref: 10750639 Clin Auton Res, 2000
- In CANP  [ **CAPNS1** ]   [ **CAPN2** ]  MMP 9 IR was found at normal sized and atrophied myofibers.  Ref: 10511442 Neuroreport, 1999
- Overall prevalence of PANP, CANP  [ **CAPNS1** ]   [ **CAPN2** ] , and SNP were 66%, 15%, and 15%, respectively.  Ref: 10750639 Clin Auton Res, 2000
- Since calcium activated neutral proteinase  [ **CAPNS1** ]   [ **CAPN2** ]  calpain is present in the central nervous system CNS and degrades myelin proteins, this endopeptidase has been suggested to play a role in myelin destruction in demyelinating diseases such as multiple sclerosis MS.  Ref: 9518658 Brain Res, 1998
- To investigate the potential therapeutic value of ANP gene delivery on salt induced stroke and cerebrovascular disorders, an adenovirus harboring the human ANP gene Ad.RSV cANP  [ **CAPNS1** ]   [ **CAPN2** ]  was injected into Dahl SS rats on a high salt diet.  Ref: 9931108 Hypertension, 1999
- The selective NPR C receptor agonist, cANP  [ **CAPNS1** ]   [ **CAPN2** ]  4 23, mimicked ANF response in a dose dependent fashion.  Ref: 12829435 Am J Physiol Gastrointest Liver Physiol< MedlineTA>Am J Physiol Gastrointest Liver Physiol, 1999
- The absence of both mu and m calpain  [ **CAPN2** ]  activity, caused by disruption of the capn4  [ **CAPNS1** ]  gene in mice, retarded migration, and disrupted the cytoskeleton, both in primary capn4  [ **CAPNS1** ]  embryonic fibroblasts mEF and in capn4  [ **CAPNS1** ]  mEF immortalized with SV40 large T antigen TAg.  Ref: 12237117 Biochem Biophys Res Commun, 2002
- In reconstitution studies, VIP, cANP  [ **CAPNS1** ]   [ **CAPN2** ]  4 23, and guanosine 5 O 3 thiotriphosphate stimulated NOS activity in membranes of COS 1 cells cotransfected with NPR C and eNOS.  Ref: 9843699 Am J Physiol, 1998
- Since calcium activated neutral proteinase  [ **CAPNS1** ]   [ **CAPN2** ]  calpain activity and expression are significantly increased in activated glial inflammatory cells in the central nervous system of animals with autoimmune demyelinating diseases, this enzyme may also play a role in peripheral organ systems in these diseases.  Ref: 10496171 J Neuroimmunol, 1999
- PANP, CANP  [ **CAPNS1** ]   [ **CAPN2** ] , and SNP were assessed by standardized test procedures.  Ref: 10750639 Clin Auton Res, 2000
- The following single strand conformation polymorphism analysis revealed a pronounced polymorphism of exon 4 of the CANP  [ **CAPNS1** ]   [ **CAPN2** ]  3 gene in 14 out of 19 patients with LGMD.  Ref: 10687099 Genetika, 1999
- Calpain calcium activated neutral proteinase  [ **CAPNS1** ]   [ **CAPN2** ]  degrades myelin proteins at physiological pH and is found in glial and inflammatory cells involved in demyelination.  Ref: 9820787 Exp Eye Res, 1998
- The remaining ANP binding sites can be occupied with the NPR C selective ligand cANP  [ **CAPNS1** ]   [ **CAPN2** ] .  Ref: 9546616 Mol Cell Biochem, 1998
- Patients with CANP  [ **CAPNS1** ]   [ **CAPN2** ]  were more often in an advanced disease stage than patients without CANP  [ **CAPNS1** ]   [ **CAPN2** ]  p = 0.004.  Ref: 10750639 Clin Auton Res, 2000
- This study shows that the loss of perifused chick pineal gland activity is a complex process which seems to involve the release of calcium from intracellular stores, calmodulin and calcium activated neutral protease CANP  [ **CAPNS1** ]   [ **CAPN2** ] .  Ref: 9605476 Neurosci Lett, 1998
- 4 After the human heart cDNA library was sequentially transformed into AH109 pGBKT7 CANP  [ **CAPNS1** ]   [ **CAPN2** ] , 1000 1200 positive clones were grown on SD Trp Leu Ade His.  Ref: 12358155 Hypertens Res, 2002
- Homozygous disruption of murine Capn4  [ **CAPNS1** ]  eliminated both mu and m calpain  [ **CAPN2** ]  activities, but this did not affect survival and proliferation of cultured embryonic stem cells or embryonic fibroblasts, or the early stages of organogenesis.  Ref: 10825211 Mol Cell Biol, 2000
- Calcium activated neutral proteinase  [ **CAPNS1** ]   [ **CAPN2** ]  is believed to participate in myelin protein degradation because known calpain substrates myelin basic protein.  Ref: 9576959 Proc Natl Acad Sci U S A, 1998
- cANP  [ **CAPNS1** ]   [ **CAPN2** ]  4 23 contracted tenia muscle cells.  Ref: 10859228 Am J Physiol Gastrointest Liver Physiol, 2000
